# Systolic blood pressure and early neurological deterioration in minor stroke: A post hoc analysis of ARAMIS trial

**DOI:** 10.1111/cns.14868

**Published:** 2024-07-16

**Authors:** Yu Cui, Zi‐Ai Zhao, Jia‐Qi Wang, Si‐Qi Qiu, Xin‐Yu Shen, Ze‐Yu Li, Hai‐Zhou Hu, Hui‐Sheng Chen

**Affiliations:** ^1^ Department of Neurology General Hospital of Northern Theater Command Shenyang China

**Keywords:** acute ischemic stroke, dual antiplatelet, early neurological deterioration, intravenous alteplase, systolic blood pressure

## Abstract

**Background:**

Systolic blood pressure (SBP) was a predictor of early neurological deterioration (END) in stroke. We performed a secondary analysis of ARAMIS trial to investigate whether baseline SBP affects the effect of dual antiplatelet versus intravenous alteplase on END.

**Methods:**

This post hoc analysis included patients in the as‐treated analysis set. According to SBP at admission, patients were divided into SBP ≥140 mmHg and SBP <140 mmHg subgroups. In each subgroup, patients were further classified into dual antiplatelet and intravenous alteplase treatment groups based on study drug actually received. Primary outcome was END, defined as an increase of ≥2 in the NIHSS score from baseline within 24 h. We investigated effect of dual antiplatelet vs intravenous alteplase on END in SBP subgroups and their interaction effect with subgroups.

**Results:**

A total of 723 patients from as‐treated analysis set were included: 344 were assigned into dual antiplatelet group and 379 into intravenous alteplase group. For primary outcome, there was more treatment effect of dual antiplatelet in SBP ≥140 mmHg subgroup (adjusted RD, −5.2%; 95% CI, −8.2% to −2.3%; *p* < 0.001) and no effect in SBP <140 mmHg subgroup (adjusted RD, −0.1%; 95% CI, −8.0% to 7.7%; *p* = 0.97), but no significant interaction between subgroups was found (adjusted *p* = 0.20).

**Conclusions:**

Among patients with minor nondisabling acute ischemic stroke, dual antiplatelet may be better than alteplase with respect to preventing END within 24 h when baseline SBP ≥140 mmHg.

## Graphical abstract

A post hoc analysis of ARAMIS trial demonstrated that among patients with minor nondisabling acute ischemic stroke, dual antiplatelet was better than alteplase with respect to preventing early neurological deterioration within 24 h when baseline systolic blood pressure ≥140 mmHg.

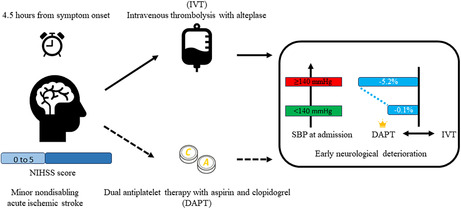

## 1 INTRODUCTION

Early neurological deterioration (END) predicts serious short‐term or long‐term outcomes for patients with acute ischemic stroke (AIS).^1^ Several factors that might contribute to the occurrence of END in AIS have been investigated.^2^, ^3^ It was well known that baseline systolic blood pressure (SBP) was significantly associated with END after AIS.^4^ For example, SBP dropping more than 20 mmHg within 3 days was associated with decreased occurrence of END in lacunar stroke patients.^5^

The Antiplatelet versus R‐tPA for Acute Mild Ischemic Stroke (ARAMIS) trial was designed and demonstrated that dual antiplatelet therapy (DAPT) was noninferior to intravenous alteplase with regard to excellent functional outcome at 90 days among minor nondisabling AIS.^6^ Interestingly, in the ARAMIS trial, DAPT was found to be associated with less END. In our recent Antiplatelet Therapy in Acute Mild to Moderate Ischemic Stroke (ATAMIS) trial,^7^ DAPT with clopidogrel plus aspirin was also proven to be superior to aspirin alone with regard to preventing the occurrence of END among acute mild to moderate ischemic stroke.

Given the association between SBP and END, it is worth exploring whether SBP at admission could affect the efficacy of DAPT on preventing the occurrence of END. Previous study investigated the association between SBP at admission and effect of DAPT on stroke recurrence in patients with minor stroke or transient ischemic attack.^8^ However, the impact of SBP at admission on the efficacy of DAPT on preventing END in minor nondisabling AIS was not well investigated. Given that elevated SBP at admission contributed to END^5^ and DAPT might be more effective than intravenous alteplase to prevent END due to the short half‐life of alteplase and lack of an antithrombotic treatment effect within 24 h after intravenous alteplase, we hypothesized that DAPT might be more effective than intravenous alteplase on preventing occurrence of END when the SBP at admission was higher. Thus, the current study was designed to test the hypothesis.

## 2 METHODS

### 2.1 Study design and participants

The current study was a post hoc analysis of the ARAMIS study and designed according to the STROBE guideline. Details on the design and protocol of ARAMIS study have been published.^6^, ^9^ In brief, ARAMIS was a multicenter, open‐label, blinded end point, noninferiority randomized clinical trial enrolling 760 patients between October 18, 2018, and April 2022, to assess the noninferiority of DAPT to intravenous alteplase in patients with minor nondisabling AIS within 4.5 h from symptom onset. The follow‐ups were completed in July 2022. Eligible patients were 18 years of age or older with AIS (NIHSS scores at admission ≤5, with ≤1 point on the NIHSS in single item scores such as vision, language, neglect, or single limb weakness and a score of 0 in the consciousness item at the time of randomization) computed tomography or magnetic resonance imaging was performed on admission to identify patients with AIS; and were enrolled up to 4.5 h after the onset of stroke symptoms. Key exclusion criteria were as follows: presence of disability in the community (modified Rankin Scale [mRS] scores ≥2) before stroke; history of intracerebral hemorrhage; or definite indication for anticoagulation. All procedures were performed according to the Declaration of Helsinki and approved by Ethics Committees of General Hospital of Northern Theater Command, and written informed consents were obtained from patients or their legally authorized representatives. The study was registered with ClinicalTrials.gov (NCT03661411). Patients from as‐treated analysis set were included in the study.

### 2.2 Procedures

Blood pressure at admission was measured twice consecutively, 1 minute apart, using a digital automatic blood pressure monitor after arriving at the emergency room or stroke unit and before any administration of antihypertensive drugs, and two measurements were averaged for record. When SBP exceeded 185 mm Hg, antihypertensive agents were given (first‐line agents: labetalol or nicardipine). Included patients were divided into two subgroups according to SBP at admission: SBP ≥140 mmHg and SBP <140 mmHg. The division point was based on previous studies reporting the significant association of SBP with END and recurrent stroke.^10^, ^11^ Furthermore, according to the drug actually received, patients in each subgroup were divided into DAPT (clopidogrel: a loading dose of 300 mg on the first day, followed by 75 mg per day for 12 ± 2 days; aspirin: 100 mg on the first day, followed by 100 mg daily for 12 ± 2 days; afterward, single or dual antiplatelet based on guidelines until 90 days) and alteplase (0.9 mg/kg [10% as a bolus, 90% infused over 1 h] to a maximum of 90 mg, followed by guideline‐based antiplatelet treatment beginning 24 h after intravenous thrombolysis) treatment groups.

Neurological status, measured with NIHSS score, was evaluated at admission and 24 h after randomization. Data on clinical characteristics were obtained at randomization. Follow‐up data were collected at 90 days after randomization. All the data were downloaded from the electronic data capture system (Shanghai Meisi Medical Technology Co., Ltd.).

### 2.3 Outcomes

The primary outcome in the current analysis was occurrence of END, compared with baseline at 24 h, defined as more than or equal to 2 NIHSS score increase. The secondary outcomes were excellent functional outcome at 90 days, defined as a mRS score of 0 to 1; favorable functional outcome at 90 days, defined as a mRS score of 0 to 2; a shift in measure of neurological function according to distribution on the mRS score at 90 days; occurrence of early neurological improvement, compared with baseline at 24 h, defined as more than or equal to 2 NIHSS score decrease; change in NIHSS score compared with baseline at 24 h; and occurrence of stroke or other vascular events and all‐cause mortality within 90 days.

The pre‐specified safety outcomes were sICH and any bleeding events that occurred during the study. sICH was defined as any evidence of bleeding on head CT associated with neurological deterioration (NIHSS ≥4‐point increase).

The assessment of baseline and follow‐up NIHSS score was done by the same assessor, who was not blinded to treatment allocation. Follow‐up at 90 days, including mRS score and stroke or other vascular events, and the judgment of adverse events were assessed through telephone or outpatient interview by trained assessors in each center who were unaware of treatment allocation or clinical details.

### 2.4 Statistical analysis

Due to the lack of a priori knowledge regarding the treatment effect of preventing END between dual antiplatelet therapy and intravenous alteplase in each SBP subgroup, we were unable to estimate a sample size accurately. The current study was based on patients in the as‐treated analysis set of ARAMIS trial. The similar characteristics of populations in as‐treated analysis set and full analysis set of ARAMIS trial addressed selection bias.^6^ Furthermore, considering imbalance between treatment groups after classification according to SBP at admission, primary analysis in the current study was adjusted analysis.

Baseline characteristics of patients and results of outcome comparison were described by different methods. For the baseline characteristics, we summarized continuous variables as medians (interquartile range [IQR]) and categorical variables as frequencies (percentages). For the outcome comparisons, we estimated absolute number of events (percentage), and absolute difference (risk difference, RD), odds ratio (OR), geometric mean ratio (GMR), hazards ratio (HR) or with their 95% confidence intervals (CIs).

First, we explored the association between SBP at admission and primary outcome. The probability of primary outcome was respectively calculated in DAPT and alteplase groups through binary logistic regression analysis, and best‐fit lines with their 95% CIs were respectively drawn according to probability and SBP at admission in each treatment group.

Second, we respectively detected the association between treatment and outcomes in SBP subgroups. Generalized linear models (GLM), which had a binomial distribution and identity link functions, were performed to generate RD or GMR. Ordinal regression analysis was performed to generate OR. Cox regression model was performed to generate HR. The two‐sided 95% CIs and adjusted *p* value were also generated with treatment effect metrics.

Accounting for baseline variables, the covariates showing difference between treatment groups with *p* values <0.1 were adjusted. To avoid non‐convergence when all adjusted covariates were introduced into the adjusted analyses simultaneously, we calculated a propensity score with treatment as the dependent variable and all adjusted covariates listed above as independent variables through a logistic regression model and then included the calculated propensity score (continuous variable) as a covariate in the model for adjusted analysis. Missing data of covariates included in the adjusted analyses were imputed through simple imputation.

Interactions of treatment effects on outcomes were assessed between SBP subgroups. The assessments of SBP at admission and effect of treatments on outcomes were conducted by GLM, ordinal regression analysis or Cox regression model with the treatment groups, SBP subgroups, and their interaction term as independent variables, and the *p*_int_ values were presented for the interaction term.

Third, we performed sensitivity analysis to address imbalanced sample size between SBP subgroups and the selection bias in analyzed population. We divided patients into four subgroups according to SBP quartiles. The dose‐response relationships between primary outcome and the SBP quartiles were examined by likelihood ratio tests for liner trend, and the *p* value for trend was presented. We also conducted a propensity score matching analysis for the primary outcome, which matched baseline patient characteristics with *p* value <0.1 between SBP subgroups under the ratio 1:1, the caliper of 0.01, and a nearest‐neighbor matching strategy. Moreover, the association of treatment effects and primary outcome was analyzed according to dichotomized SBP subgroups in full analysis set and per‐protocol analysis set population, respectively.

Fourth, a subgroup analysis of primary outcome was conducted according to seven pre‐specified factors (age [<65 years or ≥65 years], sex [female or male], history of diabetes [yes or no], NIHSS score at randomization [0.1 to 3 or 4 to 5], time from onset to treatment [≤2 h or >2 h], location of responsible vessel [anterior circulation stroke or posterior circulation stroke], and stroke etiology [undetermined cause, small artery occlusion, or large artery arteriosclerosis]) to investigate the characteristics of patients benefiting from DAPT in each SBP subgroup.

All analyses presented were exploratory, and all *p* values were nominal. Two‐sided *p* values <0.05 were considered significant. All statistical analyses were performed using the IBM SPSS software (version 26.0; SPSS Inc.) and R software (version 4.1.0; R Foundation for Statistical Computing).

## 3 RESULTS

A total of 723 patients from as‐treated analysis set of ARAMIS trial were included in the current study, including 205 in the SBP <140 mmHg subgroup and 518 in the SBP ≥140 mmHg subgroup (Figure S1). Between SBP subgroups, there were some imbalances in hypertension history and fast blood glucose at randomization beside SBP and DBP (Table 1). In the SBP <140 mmHg subgroup, there were 113 patients with DAPT and 92 patients with alteplase. Higher median onset‐to‐treatment time and more previous stroke was found in patients with DAPT. In the SBP ≥140 mmHg subgroup, there were 231 patients with DAPT and 287 patients with alteplase. Higher median age, onset‐to‐treatment time, and more hypertension were found in patients with DAPT. Details of baseline characteristics between treatment groups in each SBP subgroup are shown in Table 2.

**TABLE 1:** Baseline characteristics between baseline SBP subgroups.

	SBP < 140 mmHg (*N* = 205)	SBP ≥ 140 mmHg (*N* = 518)	*p* value
DAPT group	113 (55.1)	231 (44.6)	0.01
Age, years	64 (57–70)	65 (56–71)	0.80
Sex (F)	57 (27.8)	167 (32.2)	0.25
Current smoker	74 (36.1)	168 (32.4)	0.47
Current drinker^a^	30 (14.6)	86 (16.6)	0.83
Comorbidities^b^			
Hypertension	73 (35.6)	308 (59.5)	<0.001
Diabetes	49 (23.9)	138 (26.6)	0.45
Previous stroke^c^	41 (20.0)	119 (23.0)	0.39
Previous TIA	2 (1.0)	4 (0.8)	0.79
Blood pressure at randomization, mmHg			
Systolic	131 (125–136)	158 (149–169)	<0.001
Diastolic	80 (76–85)	90 (83–98)	<0.001
FBG at randomization, mmol/L	6.10 (5.17–7.54)	6.45 (5.52–8.41)	0.04
NIHSS score at randomization^d^	2 (1–3)	2 (1–3)	0.25
Estimated premorbid function (mRS score)^e^			
No symptoms (score, 0)	150 (73.2)	384 (74.1)	0.79
Symptoms without any disability (score, 1)	55 (26.8)	134 (25.9)	
OTT, min	175 (128–228)	183 (130–228)	0.47
Duration of hospitalization, days	8 (6–11)	8 (6–10)	0.70
Presumed stroke cause^f^			
Undetermined	132 (64.4)	316/517 (61.1)	0.81
Small artery occlusion	46 (22.4)	120/517 (23.2)	
Large artery atherosclerosis	26 (12.7)	75/517 (14.5)	
Other	1 (0.5)	4/517 (0.8)	
Cardioembolic	0 (0.0)	2/517 (0.4)	

*Note*: The data were shown with median (interquartile range) or number (percentage).

Abbreviations: DAPT, dual antiplatelet therapy; FBG, fasting blood glucose; mRS, modified Rankin Scale; NIHSS, National Institute of Health Stroke Scale; OTT, time from onset of symptom to intravenous thrombolysis or dual antiplatelet therapy; SBP, systolic blood pressure; TIA, transient ischemic attack.

^a^ Defined as consuming alcohol at least once a week within 1 year prior to the onset of the disease.

^b^ The comorbidities were based on the patient or family report.

^c^ Previous stroke included ischemic and hemorrhagic stroke.

^d^ NIHSS scores range from 0 to 42, with higher scores indicating more severe neurological deficit.

^e^ Scores on the mRS of functional disability range from 0 (no symptoms) to 6 (death).

^f^ The presumed stroke cause was classified according to the Trial of ORG10172 in Acute Stroke Treatment (TOAST) using clinical findings, brain imaging, and laboratory test results. Other causes included nonatherosclerotic vasculopathies, hypercoagulable states, and hematologic disorder.

**TABLE 2:** Baseline characteristics between treatment groups in each baseline SBP subgroup.

	SBP < 140 mmHg			SBP ≥ 140 mmHg		
	DAPT (*N* = 113)	Alteplase (*N* = 92)	*p* value	DAPT (*N* = 231)	Alteplase (N = 287)	*p* value
Age, years	65 (58–71)	63 (56–70)	0.68	66 (57–73)	63 (56–70)	0.02
Sex (F)	35 (31.0)	22 (23.9)	0.26	76 (32.9)	91 (31.7)	0.77
Current smoker	35 (31.0)	39 (42.4)	0.20	64 (27.7)	104 (36.0)	0.06
Current drinker^a^	16 (14.2)	14 (15.2)	0.11	34 (14.7)	52 (18.1)	0.43
Comorbidities^b^						
Hypertension	46 (40.7)	27 (29.3)	0.09	150 (64.9)	158 (55.1)	0.02
Diabetes	25 (22.1)	26 (26.1)	0.51	61 (26.4)	77 (26.8)	0.91
Previous stroke^c^	29 (25.7)	12 (13.0)	0.03	55 (23.8)	64 (22.3)	0.69
Previous TIA	1 (0.9)	1 (1.1)	0.88	3 (1.3)	1 (0.3)	0.22
Blood pressure at randomization, mmHg						
Systolic	131 (127–136)	131 (122–137)	0.92	158 (149–169)	159 (150–170)	0.37
Diastolic	81 (77–85)	80 (75–85)	0.45	90 (85–97)	90 (83–98)	0.53
FBG at randomization, mmol/L	5.95 (5.19–7.50)	6.20 (5.13–7.68)	0.71	6.37 (5.50–8.30)	6.50 (5.56–8.70)	0.48
NIHSS score at randomization^d^	2 (1–3)	2 (1–3)	0.30	2 (1–3)	2 (1–3)	0.50
Estimated premorbid function (mRS score)^e^						
No symptoms (score, 0)	82 (72.6)	68 (73.9)	0.83	164 (71.0)	220 (76.7)	0.14
Symptoms without any disability (score, 1)	31 (27.4)	24 (26.1)		67 (29.0)	67 (23.3)	
OTT, min	182 (136–240)	168 (121–204)	0.048	191 (138–235)	170 (125–220)	0.005
Duration of hospitalization, d	8 (6–11)	8 (6–11)	0.74	8 (6–10)	8 (6–10)	0.43
Presumed stroke cause^f^						
Undetermined	70 (61.9)	62 (67.4)	0.46	153 (66.2)	163/286 (57.0)	0.27
Small artery occlusion	26 (23.0)	20 (21.7)		48 (20.8)	72/286 (25.2)	
Large artery atherosclerosis	17 (15.0)	9 (9.8)		27 (11.1)	48/286 (16.8)	
Other	0 (0.0)	1 (1.1)		2 (0.9)	2/286 (0.7)	
Cardioembolic	0 (0.0)	0 (0.0)		1 (0.4)	1/286 (0.3)	

*Note:* The data were shown with median (interquartile range) or number (percentage).

Abbreviations: DAPT, dual antiplatelet therapy; FBG, fasting blood glucose; mRS, modified Rankin Scale; NIHSS, National Institute of Health Stroke Scale; OTT, time from onset of symptom to intravenous thrombolysis or dual antiplatelet therapy; SBP, systolic blood pressure; TIA, transient ischemic attack.

^a^ Defined as consuming alcohol at least once a week within 1 year prior to the onset of the disease.

^b^ The comorbidities were based on the patient or family report.

^c^ Previous stroke included ischemic and hemorrhagic stroke.

^d^ NIHSS scores range from 0 to 42, with higher scores indicating more severe neurological deficit.

^e^ Scores on the mRS of functional disability range from 0 (no symptoms) to 6 (death).

^f^ The presumed stroke cause was classified according to the Trial of ORG10172 in Acute Stroke Treatment (TOAST) using clinical findings, brain imaging, and laboratory test results. Other causes included nonatherosclerotic vasculopathies, hypercoagulable states, and hematologic disorder.

**FIGURE 1:**
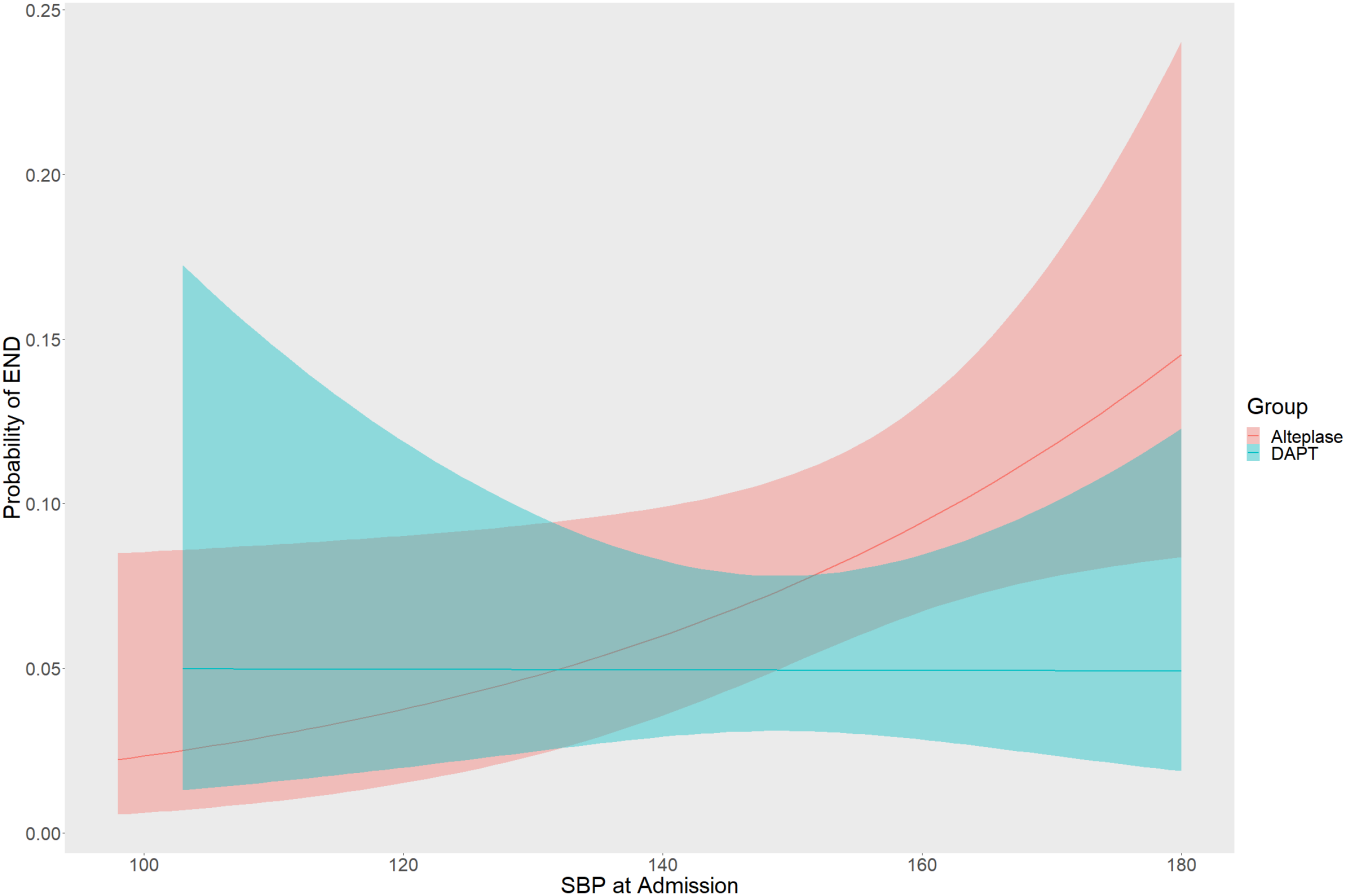
Probability of END according to baseline SBP. DAPT, dual antiplatelet therapy; END, early neurological deterioration; SBP, systolic blood pressure.

First, as a continuous variable, we detected the association of treatment effect on END with SBP at admission. The probability of END at 24 h increased as SBP at admission increased in alteplase group. Compared with alteplase group, the probability was lower in the DAPT group after SBP >140 mmHg and gap of probability between groups became large as SBP at admission increased (Figure 1).

Second, we detected treatment effect on outcomes with dichotomized SBP at admission. The comparisons of outcomes between DAPT and alteplase in each subgroup are shown in Table 3. The proportion of patients with END in the DAPT group and alteplase group was 5.3% versus 4.3% in the SBP <140 mmHg subgroup and 4.8% versus 9.8% in the SBP ≥140 mmHg subgroup. Compared with alteplase, DAPT reduced likelihood of END in SBP ≥140 mmHg subgroup (adjusted RD, −5.2%; 95% CI, −8.2% to −2.3%; *p* < 0.001). However, there was no significant interaction between SBP subgroups. For the secondary and safety outcomes, no significant differences between treatment groups were found in any subgroup. No significant interaction for any secondary outcome was also found between SBP subgroups.

**TABLE 3:** Outcomes comparison between treatments groups according to baseline SBP.

Outcomes	Subgroups^a^	No. (%) of events or median difference		Unadjusted		Adjusted^b^		*P*_int_ value
		DAPT	Alteplase	Treatment difference (95% CI)	*p* value	Treatment difference (95% CI)	*p* value	
Primary outcome								
END within 24 h^c^	SBP <140 mmHg	6/113 (5.3)	4/92 (4.3)	1.0 (−4.9 to 6.8)	0.75	−0.1 (−8.0 to 7.7)	0.97	0.20
	SBP ≥140 mmHg	11/231 (4.8)	28/287 (9.8)	−5.0 (−9.4 to −0.6)	0.03	−5.2 (−8.2 to −2.3)	<0.001	
Secondary outcomes								
mRS 0–1 at 90 days^d^, ^e^	SBP <140 mmHg	105/113 (93.8)	86/92 (93.5)	0.3 (−6.5 to 7.0)	0.94	0.8 (−3.7 to 5.4)	0.73	0.69
	SBP ≥140 mmHg	215/231 (93.5)	260/287 (91.2)	2.3 (−2.3 to 6.8)	0.34	1.5 (−1.5 to 4.6)	0.33	
mRS 0–2 at 90 days^d^, ^e^	SBP <140 mmHg	106/113 (94.6)	87/92 (94.6)	0.1 (−6.2 to 6.3)	0.98	0.4 (−3.8 to 4.7)	0.84	0.84
	SBP ≥140 mmHg	222/231 (96.5)	273/287 (95.8)	0.7 (−2.6 to 4.1)	0.67	0.2 (−2.0 to 2.4)	0.84	
mRS distribution at 90 days^d^, ^f^	SBP <140 mmHg	–	–	0.70 (0.37 to 1.34)	0.28	0.72 (0.37 to 1.38)	0.32	0.21
	SBP ≥140 mmHg	–	–	1.14 (0.77 to 1.69)	0.52	1.07 (0.82 to 1.40)	0.61	
Early neurological improvement within 24 h^g^	SBP <140 mmHg	16/113 (14.2)	29/92 (31.5)	−17.4 (−28.8 to −5.9)	0.003	−15.4 (−27.1 to −3.6)	0.01	0.23
	SBP ≥140 mmHg	31/231 (13.4)	60/287 (20.9)	−7.5 (−13.9 to −1.0)	0.02	−9.0 (−13.3 to −4.7)	<0.001	
Change in NIHSS score at 24 h^h^	SBP <140 mmHg	0.00 (−0.46 to 0.00)	−0.41 (−0.69 to 0.00)	−0.19 (−0.33 to −0.05)	0.006	−0.17 (−0.31 to −0.03)	0.02	0.17
	SBP ≥140 mmHg	0.00 (−0.41 to 0.00)	0.00 (−0.69 to 0.00)	−0.07 (−0.16 to 0.03)	0.18	−0.09 (−0.16 to 0.03)	0.007	
Stroke or other vascular events within 90 days^i^	SBP <140 mmHg	0/112 (0.0)	0/92 (0.0)	NA	NA	NA	NA	0.99
	SBP ≥140 mmHg	1/230 (0.4)	2/285 (0.7)	0.62 (0.06 to 6.80)	0.69	0.55 (0.05 to 6.45)	0.64	
All‐cause death at 90 days^e^	SBP <140 mmHg	1/112 (0.9)	1/92 (1.1)	−0.2 (−2.9 to 2.5)	0.89	−0.4 (−2.2 to 1.4)	0.69	0.92
	SBP ≥140 mmHg	1/230 (0.4)	2/285 (0.7)	−0.3 (−1.6 to 1.0)	0.69	−0.1 (−1.0 to 0.7)	0.75	
Safety outcomes								
Symptomatic intracerebral hemorrhage^e^, ^j^	SBP <140 mmHg	0/113 (0.0)	0/92 (0.0)	NA	NA	NA	NA	N/A
	SBP ≥140 mmHg	0/231 (0.0)	4/287 (1.4)	NA	<0.001	NA	<0.001	
Any bleeding events	SBP <140 mmHg	2/113 (1.8)	3/92 (3.3)	−1.5 (−5.9 to 2.9)	0.50	−1.3 (−4.1 to 1.5)	0.35	N/A
	SBP ≥140 mmHg	0/231 (0.0)	20/287 (7.0)	N/A	<0.001	N/A	<0.001	

*Note*: *p*_int_ value means the *p* value for interaction.

Abbreviations: CI, confidence interval; DAPT, dual antiplatelet therapy; END, early neurological deterioration; mRS, modified Rankin Scale; N/A, not applicable; NIHSS, National Institute of Health Stroke Scale; and SBP, systolic blood pressure.

^a^ There were 205 patients in the SBP <140 mmHg subgroup and 518 patients in the SBP ≥140 mmHg subgroup.

^b^ Adjusted for covariates compared between DAPT and alteplase treatment groups with *p* value <0.1 in each subgroup.

^c^ END was defined as an increase between baseline and 24 h of 2 on the NIHSS score, but not as a result of cerebral hemorrhage.

^d^ mRS scores range from 0 to 6: 0 = no symptoms, 1 = symptoms without clinically significant disability, 2 = slight disability, 3 = moderate disability, 4 = moderately severe disability, 5 = severe disability, and 6 = death.

^e^ Calculated using generalized linear model and presented by risk difference.

^f^ Calculated using ordinal regression analysis and presented by odds ratio.

^g^ Early neurological improvement was defined as a decrease between baseline and 24 h of 2 on the NIHSS score.

^h^ NIHSS scores range from 0 to 42, with higher scores indicating greater stroke severity. Log (NIHSS+1) was analyzed using generalized linear model and presented by geometric mean ratio.

^i^ Calculated using Cox regression model and presented by hazard ratio.

^j^ Symptomatic intracerebral hemorrhage was defined as any evidence of bleeding on head CT associated with neurological deterioration (NIHSS ≥4‐point increase).

**FIGURE 2:**
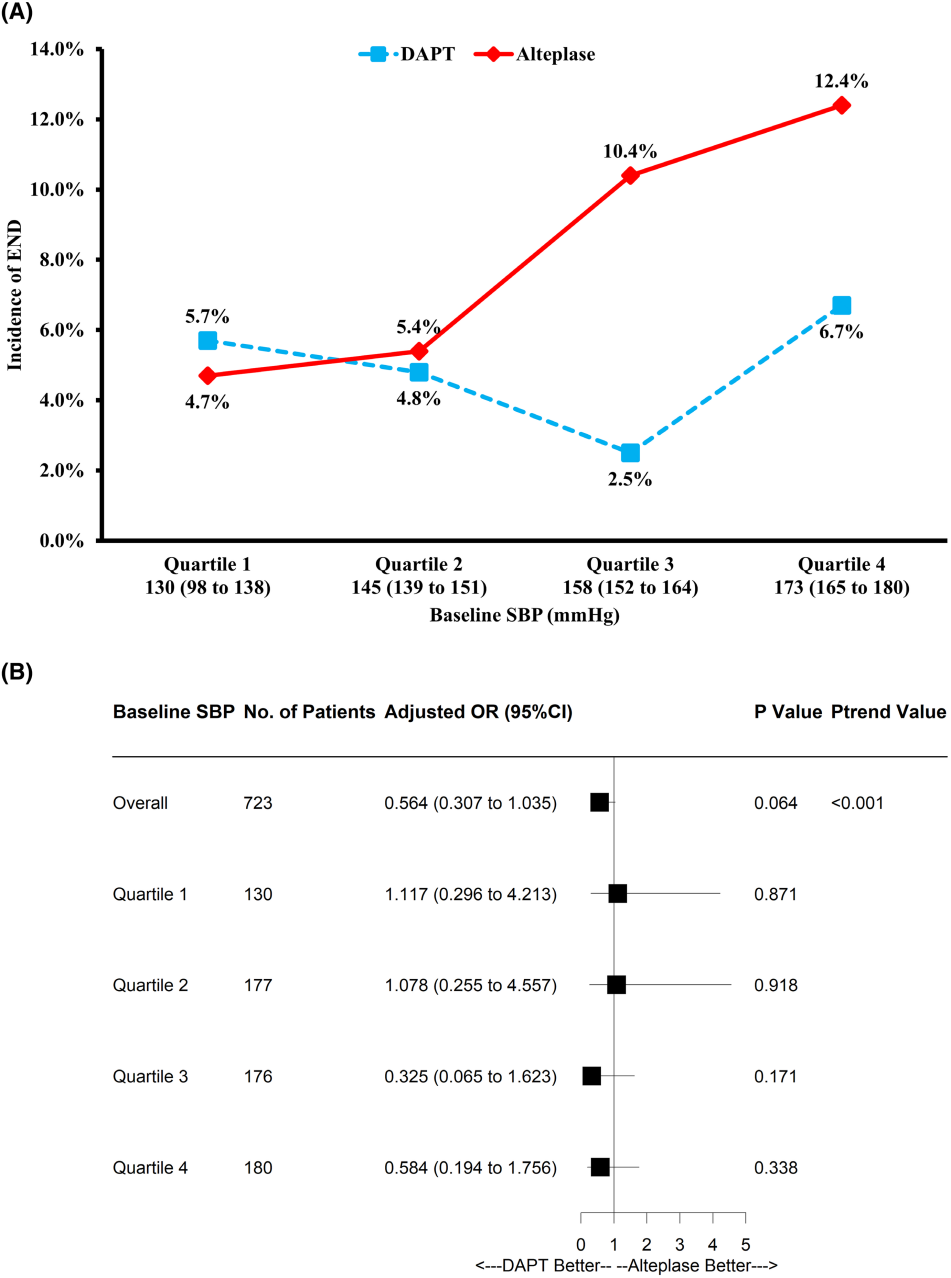
Proportion (A) and likelihood (B) of END in DAPT versus alteplase according to quartiles of baseline SBP. For subcategories, black squares represent the odds ratio and horizontal lines represent the 95% confidence interval. DAPT, dual antiplatelet therapy; END, early neurological deterioration; SBP, systolic blood pressure.

Third, the treatment effects among four subgroups according to SBP quartiles were determined as a sensitivity analysis. There were 190 patients in the Q1 130 (98–138) mmHg, 177 patients in the Q2 145 (139–151) mmHg, 176 patients in the Q3 158 (152–164) mmHg, and 180 patients in the Q4 173 (165–180) mmHg SBP subgroups. The results of END within 24 h between treatment groups in each SBP quartile subgroup are shown in Figure 2. The odds for developing END increased in a dose‐dependent manner as SBP at admission increased (*p* value for trend <0.001). Moreover, similar results were found in population from propensity score matching analysis set, full analysis set, and per‐protocol analysis set with regard to the primary outcome (Table S5).

Fourth, in the subgroup analysis, there was not any significant interaction between SBP at admission and the effect of DAPT on preventing END in each subgroup of seven pre‐specified factors (Figure S2).

## 4 DISCUSSION

In this post hoc analysis of ARAMIS trial, we explored the effect of systolic blood pressure at admission on clinical outcomes after stroke in patients receiving DAPT. The results showed that compared with alteplase, DAPT significantly reduced risk of END within 24 h in AIS patients with SBP ≥140 mmHg at admission. Furthermore, the probability of END increased as SBP at admission increased in alteplase group while it kept constant in DAPT group.

Results of this post hoc analysis demonstrated that patients with higher SBP at admission may benefit from DAPT with respect to preventing END. As short half‐life time of alteplase contributed to lacking persistent antithrombotic effect within 24 h after intravenous thrombolysis, patients with AIS would have a high risk of END after thrombolysis without the use of antiplatelet. Conversely, DAPT could prevent occurrence of END within the 24 h after stroke onset, during which stroke progression was the main cause of END.^12^ Although DAPT was found associated with reduced risk of END in full analysis set, it was unexpected to find the association was not statistically significant in as‐treated analysis set.^6^ It was worth exploring what patients may have a less risk of END after receiving actual agents.

Given that blood pressure was previously reported to be a predictor of END in AIS, we investigated the treatment effects on preventing END according to different baseline SBP levels. As a continuous variable, higher SBP at admission was found to be associated with higher probability of END in patients treated with intravenous alteplase than DAPT. Similar results were also found when analyzing SBP at admission as dichotomized variable or quartiles. The likelihood of END in patients receiving intravenous alteplase increased with higher SBP at admission, which was reported in previous meta‐analysis.^13^ This may be explained by higher pretreatment SBP levels were associated with poor recanalization in patients with AIS receiving intravenous alteplase treatment,^14^ which contributed to post‐thrombolytic END in AIS.^15^ Conversely, the proportion of END in patients receiving DAPT seemed to persist consistent across different baseline SBP levels. As poor cerebral hemodynamic reserve and absence of collateral blood supply were associated with END,^16^, ^17^ improving cerebral perfusion may be effective to preventing END. Given that higher blood pressure improved cerebral perfusion after AIS,^18^ we inferred that the antithrombotic treatment of DAPT contributed to the effect. Thus, higher SBP at admission played different roles in patients receiving intravenous alteplase and DAPT treatments. Generally, patients may benefit more from DAPT than intravenous alteplase with respect to reducing risk of END at 24 h when SBP at admission was higher than 140 mmHg.

For the secondary outcome, the alteplase group showed higher likelihood of early neurological improvement and decrease in NIHSS score at 24 h than DAPT in any SBP subgroups. These were similar with those in the ARAMIS trial. Furthermore, the higher proportion of early neurological improvement in the alteplase group may partially explain the similar proportion of excellent and independent functional outcomes at 90 days between DAPT and alteplase groups. Moreover, for the safety outcome, DAPT showed lower incidence of bleeding events compared with intravenous alteplase.

In the subgroup analysis, we did not find any significant interaction between SBP at admission and the efficacy of DAPT on preventing the occurrence of END in SBP subgroups. Previous studies demonstrated that sexual dimorphism has been increasingly recognized in healthy population and patients as an important factors that might modify the response to therapies in neurovascular disorders.^19^, ^20^, ^21^ However, the influence from sexual dimorphism was not found in the current study. On one hand, the result may be partially attributed to relatively unbalanced sample size between male and female patients in the analysis. On the other hand, relatively lower occurrence of END in the female patients (4.5%) compared with male patients (7.8%) may weaken the different treatment effect of DAPT between two populations. Thus, whether sexual dimorphism influenced the efficacy of DAPT on preventing END needs further investigation.

The main strength of this study was the first attempt to investigate the effect of DAPT in minor nondisabling AIS with different SBP at admission and found that DAPT may improve early functional outcome in patients with higher SBP at admission. However, we admitted there were several limitations in the current study. First, statistical power would be affected by imbalanced sample size in each SBP subgroup. Although we performed sensitivity analysis to address the bias between SBP subgroups to some extent, lower proportion of patients in each subgroup still affected the power. Thus, this finding warrants further investigation in a cohort with adequate and balanced sample size. Second, patients with SBP >180 mmHg were excluded from the ARAMIS trial, and hence, no data were available for those with severe hypertension at admission. Third, in the current study, we only detected the association between SBP and treatment effect on END. More parameters of blood pressure, such as blood pressure variability or mean artery pressure, should be investigated to validate the association. Fourth, we did not measure the recanalization status after intravenous alteplase, which may partly contribute to explain the findings. Fifth, the generalizability of the results would need to be validated in other cohorts, particularly in a non‐Chinese population. Finally, these findings should be interpreted with caution due to exploratory nature of post hoc analysis and warrant confirmation.

## 5 CONCLUSION

With respect to preventing END within 24 h, DAPT may be more beneficial than intravenous alteplase in patients presenting with minor nondisabling AIS and SBP at admission ≥140 mmHg.

## AUTHOR CONTRIBUTIONS

HSC contributed to conception and design of the study and critically revised the manuscript; YC contributed to analysis and interpretation of data and drafting the text; ZAZ contributed to acquisition of data; SQQ, XYS, ZYL, and HZH contributed to data collection; and JQW contributed to preparing the figures.

## FUNDING INFORMATION

This study was supported by grants from the Science and Technology Project Plan of Liaoning Province (2022JH2/101500020). The funders of the study had no role in the study design, data collection, data analysis, data interpretation, or writing of the report.

## CONFLICT OF INTEREST STATEMENT

The authors report no competing interests.

## Supporting information


Data S1:

## Data Availability

Data that support findings of this study are available from the corresponding author on reasonable request.
