# Supplementary material for: Systolic blood pressure and early neurological deterioration in minor stroke: A post hoc analysis of ARAMIS trial
Source: CNS Neurosci Ther. 2024 Jul 16;30(7):e14868. doi: 10.1111/cns.14868 (PMC11252017; doi:10.1111/cns.14868)
Supplement: Supplementary file 1 — Data S1: [file CNS-30-e14868-s001.docx]

**Supplementary Materials**

**“Systolic blood pressure and early neurological deterioration in minor stroke**

**A post hoc analysis of ARAMIS trial”**

**Yu Cui,** **Ph.D.; Zi-Ai Zhao, M.D.; Jia-Qi Wang, M.M.; Si-Qi Qiu, B.S.M.; Xin-Yu Shen, B.S.M.; Ze-Yu Li, B.S.M.; Hai-Zhou Hu, B.S.M.; Hui-Sheng Chen, M.D., Ph.D.**

**Content**

[**Table S1 Baseline Characteristics between Treatment Groups in Quartile 1 of Baseline SBP. 2**](#_Toc166009825)

[**Table S2 Baseline Characteristics between Treatment Groups in Quartile 2 of Baseline SBP. 3**](#_Toc166009826)

[**Table S3 Baseline Characteristics between Treatment Groups in Quartile 3 of Baseline SBP. 4**](#_Toc166009827)

[**Table S4 Baseline Characteristics between Treatment Groups in Quartile 4 of Baseline SBP. 5**](#_Toc166009828)

[**Table S5 Primary Outcome Comparison between Treatments Groups According to Baseline SBP in Different Populations. 6**](#_Toc166009829)

[**Figure S1 Flow Chart. 7**](#_Toc166009830)

[**Figure S2 Subgroup Analysis. 8**](#_Toc166009831)

# **Table S1 Baseline Characteristics between Treatment Groups in Quartile 1 of Baseline SBP.**

|  | **DAPT (N=105)** | **Alteplase (N=85)** | ***P* Value** |
| --- | --- | --- | --- |
| Age, y | 65 (59-71) | 63 (56-70) | 0.65 |
| Sex (F) | 33 (31.4) | 21 (24.7) | 0.31 |
| Current smoker | 30 (28.6) | 33 (38.8) | 0.27 |
| Current drinker ^a^ | 14 (13.3) | 13 (15.3) | 0.14 |
| Comorbidities ^b^ | | | |
| Hypertension | 41 (39.0) | 24 (28.2) | 0.12 |
| Diabetes | 24 (22.9) | 21 (24.7) | 0.77 |
| Previous stroke ^c^ | 25 (23.8) | 11 (12.9) | 0.06 |
| Previous TIA | 1 (1.0) | 1 (1.2) | 0.88 |
| Blood pressure at randomization, mmHg | | | |
| Systolic | 131 (126-134) | 130 (121-136) | 0.84 |
| Diastolic | 81 (78-85) | 80 (75-84) | 0.38 |
| FBG at randomization, mmol/L | 5.99 (5.27-7.51) | 6.20 (5.11-7.67) | 0.93 |
| NIHSS score at randomization ^d^ | 2 (1-3) | 2 (1-3) | 0.38 |
| Estimated premorbid function (mRS score) ^e^ | | | |
| No symptoms (score, 0) | 76 (72.4) | 64 (75.3) | 0.65 |
| Symptoms without any disability (score, 1) | 29 (27.6) | 21 (24.7) |  |
| OTT, min | 178 (129-238) | 165 (115-203) | 0.048* |
| Duration of hospitalization, d | 8 (6-11) | 8 (6-11) | 0.42 |
| Presumed stroke cause ^f^ | | | |
| Undetermined | 65 (61.9) | 196 (68.8) | 0.52 |
| Small artery occlusion | 24 (22.9) | 57 (20.0) |  |
| Large artery atherosclerosis | 16 (15.2) | 13 (4.6) |  |
| Other | 0 (0.0) | 18 (6.3) |  |
| Cardioembolic | 0 (0.0) | 0 (0.0) |  |

The data was shown with median (interquartile range) or number (percentage). Abbreviation: DAPT, dual antiplatelet therapy; FBG, fasting blood glucose; mRS, modified Rankin Scale; NIHSS, National Institute of Health Stroke Scale; OTT, time from onset of symptom to intravenous thrombolysis or dual antiplatelet therapy; SBP, systolic blood pressure; TIA, transient ischemic attack.

^a^ Defined as consuming alcohol at least once a week within 1 year prior to the onset of the disease.

^b^ The comorbidities were based on the patient or family report.

^c^ Previous stroke included ischemic and hemorrhagic stroke.

^d^ NIHSS scores range from 0 to 42, with higher scores indicating more severe neurological deficit.

^e^ Scores on the mRS of functional disability range from 0 (no symptoms) to 6 (death).

^f^ The presumed stroke cause was classified according to the Trial of ORG10172 in Acute Stroke Treatment (TOAST) using clinical findings, brain imaging, and laboratory test results. Other causes included nonatherosclerotic vasculopathies, hypercoagulable states, and hematologic disorder.

* *P* value <0.05.

# **Table S2 Baseline Characteristics between Treatment Groups in Quartile 2 of Baseline SBP.**

|  | **DAPT (N=84)** | **Alteplase (N=93)** | ***P* Value** |
| --- | --- | --- | --- |
| Age, y | 65 (56-75) | 63 (54-70) | 0.16 |
| Sex (F) | 24 (28.6) | 29 (31.2) | 0.71 |
| Current smoker | 20 (23.8) | 38 (40.9) | 0.045* |
| Current drinker ^a^ | 12 (14.3) | 20 (21.5) | 0.63 |
| Comorbidities ^b^ | | | |
| Hypertension | 54 (64.3) | 43 (46.2) | 0.02* |
| Diabetes | 24 (28.6) | 22 (23.7) | 0.46 |
| Previous stroke ^c^ | 22 (26.2) | 16 (17.2) | 0.15 |
| Previous TIA | 1 (1.2) | 0 (0.0) | 0.29 |
| Blood pressure at randomization, mmHg | | | |
| Systolic | 144 (140-148) | 145 (141-149) | 0.52 |
| Diastolic | 87 (81-90) | 86 (80-93) | 0.86 |
| FBG at randomization, mmol/L | 6.50 (5.40-9.22) | 6.76 (5.38-8.15) | 0.98 |
| NIHSS score at randomization ^d^ | 2 (1-3) | 2 (1-3) | 0.31 |
| Estimated premorbid function (mRS score) ^e^ | | | |
| No symptoms (score, 0) | 56 (66.7) | 66 (71.0) | 0.54 |
| Symptoms without any disability (score, 1) | 28 (33.3) | 27 (29.0) |  |
| OTT, min | 205 (149-239) | 183 (139-234) | 0.15 |
| Duration of hospitalization, d | 9 (7-11) | 7 (5-9) | 0.003** |
| Presumed stroke cause ^f^ | | | |
| Undetermined | 50/84 (59.5) | 65/92 (70.7) | 0.40 |
| Small artery occlusion | 22/84 (26.2) | 19/92 (20.7) |  |
| Large artery atherosclerosis | 10/84 (11.9) | 8/92 (8.7) |  |
| Other | 1/84 (1.2) | 0/92 (0.0) |  |
| Cardioembolic | 1/84 (1.2) | 0/92 (0.0) |  |

The data was shown with median (interquartile range) or number (percentage). Abbreviation: DAPT, dual antiplatelet therapy; FBG, fasting blood glucose; mRS, modified Rankin Scale; NIHSS, National Institute of Health Stroke Scale; OTT, time from onset of symptom to intravenous thrombolysis or dual antiplatelet therapy; SBP, systolic blood pressure; TIA, transient ischemic attack.

^a^ Defined as consuming alcohol at least once a week within 1 year prior to the onset of the disease.

^b^ The comorbidities were based on the patient or family report.

^c^ Previous stroke included ischemic and hemorrhagic stroke.

^d^ NIHSS scores range from 0 to 42, with higher scores indicating more severe neurological deficit.

^e^ Scores on the mRS of functional disability range from 0 (no symptoms) to 6 (death).

^f^ The presumed stroke cause was classified according to the Trial of ORG10172 in Acute Stroke Treatment (TOAST) using clinical findings, brain imaging, and laboratory test results. Other causes included nonatherosclerotic vasculopathies, hypercoagulable states, and hematologic disorder.

* *P* value <0.05, ** *P* value <0.01, and *** *P* value <0.001.

# **Table S3 Baseline Characteristics between Treatment Groups in Quartile 3 of Baseline SBP.**

|  | **DAPT (N=80)** | **Alteplase (N=96)** | ***P* Value** |
| --- | --- | --- | --- |
| Age, y | 66 (58-73) | 63 (56-71) | 0.17 |
| Sex (F) | 25 (31.3) | 26 (27.1) | 0.54 |
| Current smoker | 22 (27.5) | 42 (43.8) | 0.05 |
| Current drinker ^a^ | 9 (11.3) | 21 (21.9) | 0.27 |
| Comorbidities ^b^ | | | |
| Hypertension | 50 (62.5) | 51 (53.1) | 0.21 |
| Diabetes | 15 (18.8) | 30 (31.3) | 0.06 |
| Previous stroke ^c^ | 17 (21.3) | 22 (22.9) | 0.79 |
| Previous TIA | 1 (1.3) | 1 (1.0) | 0.90 |
| Blood pressure at randomization, mmHg | | | |
| Systolic | 174 (170-177) | 173 (168-178) | 0.69 |
| Diastolic | 96 (90-102) | 95 (89-101) | 0.82 |
| FBG at randomization, mmol/L | 6.39 (5.53-8.18) | 6.45 (5.61-8.80) | 0.59 |
| NIHSS score at randomization ^d^ | 2 (1-3) | 3 (2-4) | 0.001** |
| Estimated premorbid function (mRS score) ^e^ | | | |
| No symptoms (score, 0) | 61 (76.3) | 76 (79.2) | 0.64 |
| Symptoms without any disability (score, 1) | 19 (23.8) | 20 (20.8) |  |
| OTT, min | 187 (144-240) | 160 (120-207) | 0.003** |
| Duration of hospitalization, d | 9 (7-11) | 8 (6-11) | 0.47 |
| Presumed stroke cause ^f^ | | | |
| Undetermined | 57 (71.3) | 54 (51.4) | 0.16 |
| Small artery occlusion | 10 (12.5) | 33 (31.4) |  |
| Large artery atherosclerosis | 6 (8.0) | 15 (14.3) |  |
| Other | 0 (0.0) | 2 (1.9) |  |
| Cardioembolic | 0 (0.0) | 1 (1.0) |  |

The data was shown with median (interquartile range) or number (percentage). Abbreviation: DAPT, dual antiplatelet therapy; FBG, fasting blood glucose; mRS, modified Rankin Scale; NIHSS, National Institute of Health Stroke Scale; OTT, time from onset of symptom to intravenous thrombolysis or dual antiplatelet therapy; SBP, systolic blood pressure; TIA, transient ischemic attack.

^a^ Defined as consuming alcohol at least once a week within 1 year prior to the onset of the disease.

^b^ The comorbidities were based on the patient or family report.

^c^ Previous stroke included ischemic and hemorrhagic stroke.

^d^ NIHSS scores range from 0 to 42, with higher scores indicating more severe neurological deficit.

^e^ Scores on the mRS of functional disability range from 0 (no symptoms) to 6 (death).

^f^ The presumed stroke cause was classified according to the Trial of ORG10172 in Acute Stroke Treatment (TOAST) using clinical findings, brain imaging, and laboratory test results. Other causes included nonatherosclerotic vasculopathies, hypercoagulable states, and hematologic disorder.

* *P* value <0.05, ** *P* value <0.01, and *** *P* value <0.001.

# **Table S4 Baseline Characteristics between Treatment Groups in Quartile 4 of Baseline SBP.**

|  | **DAPT (N=75)** | **Alteplase (N=105)** | ***P* Value** |
| --- | --- | --- | --- |
| Age, y | 65 (59-71) | 63 (56-70) | 0.65 |
| Sex (F) | 29 (38.7) | 37 (35.2) | 0.64 |
| Current smoker | 27 (36.0) | 30 (28.6) | 0.41 |
| Current drinker ^a^ | 15 (20.0) | 12 (11.4) | 0.37 |
| Comorbidities ^b^ | | | |
| Hypertension | 51 (68.0) | 67 (63.8) | 0.56 |
| Diabetes | 23 (30.7) | 28 (26.7) | 0.56 |
| Previous stroke ^c^ | 20 (26.7) | 27 (25.7) | 0.89 |
| Previous TIA | 1 (1.3) | 0 (0.0) | 0.24 |
| Blood pressure at randomization, mmHg | | | |
| Systolic | 131 (126-134) | 130 (121-136) | 0.84 |
| Diastolic | 81 (78-85) | 80 (75-84) | 0.38 |
| FBG at randomization, mmol/L | 5.99 (5.27-7.51) | 6.20 (5.11-7.67) | 0.93 |
| NIHSS score at randomization ^d^ | 2 (1-3) | 2 (1-3) | 0.38 |
| Estimated premorbid function (mRS score) ^e^ | | | |
| No symptoms (score, 0) | 53 (70.7) | 82 (78.1) | 0.26 |
| Symptoms without any disability (score, 1) | 22 (29.3) | 23 (21.9) |  |
| OTT, min | 178 (129-238) | 165 (115-203) | 0.048* |
| Duration of hospitalization, d | 8 (6-11) | 8 (6-11) | 0.42 |
| Presumed stroke cause ^f^ | | | |
| Undetermined | 51 (68.0) | 196 (68.8) | 0.52 |
| Small artery occlusion | 18 (24.0) | 57 (20.0) |  |
| Large artery atherosclerosis | 16 (15.2) | 13 (4.6) |  |
| Other | 0 (0.0) | 18 (6.3) |  |
| Cardioembolic | 0 (0.0) | 0 (0.0) |  |

The data was shown with median (interquartile range) or number (percentage). Abbreviation: DAPT, dual antiplatelet therapy; FBG, fasting blood glucose; mRS, modified Rankin Scale; NIHSS, National Institute of Health Stroke Scale; OTT, time from onset of symptom to intravenous thrombolysis or dual antiplatelet therapy; SBP, systolic blood pressure; TIA, transient ischemic attack.

^a^ Defined as consuming alcohol at least once a week within 1 year prior to the onset of the disease.

^b^ The comorbidities were based on the patient or family report.

^c^ Previous stroke included ischemic and hemorrhagic stroke.

^d^ NIHSS scores range from 0 to 42, with higher scores indicating more severe neurological deficit.

^e^ Scores on the mRS of functional disability range from 0 (no symptoms) to 6 (death).

^f^ The presumed stroke cause was classified according to the Trial of ORG10172 in Acute Stroke Treatment (TOAST) using clinical findings, brain imaging, and laboratory test results. Other causes included nonatherosclerotic vasculopathies, hypercoagulable states, and hematologic disorder.

* *P* value <0.05, ** *P* value <0.01, and *** *P* value <0.001.

# **Table S5 Primary Outcome Comparison between Treatments Groups According to Baseline SBP in Different Populations.**

| **Outcomes** | **Population** | **Subgroups** ^a^ | **No.(%) of events** | | **Unadjusted** | | **Adjusted** ^b^ | | ***P*_int_ value** |
| --- | --- | --- | --- | --- | --- | --- | --- | --- | --- |
|  |  |  | **DAPT** | **Alteplase** | **Treatment**  **difference (95% CI)** | ***P***  **value** | **Treatment**  **difference (95% CI)** | ***P***  **value** |  |
| **Primary outcome** | | | | | | | | | |
| END within 24 h ^c^ | Propensity score matching analysis set | SBP <140 mmHg | 4/97 (4.1) | 3/82 (3.7) | 0.5 (-5.2 to 6.1) | 0.87 | NA | NA | 0.18 |
|  |  | SBP ≥140 mmHg | 5/98 (5.1) | 12/81 (14.8) | -9.7 (-18.6 to -0.8) | 0.03* | NA | NA |  |
|  | Full analysis set | SBP <140 mmHg | 6/110 (5.5) | 4/94 (4.3) | 1.2 (-4.7 to 7.1) | 0.69 | 1.1 (-2.6 to 4.8) | 0.58 | 0.02* |
|  |  | SBP ≥140 mmHg | 11/259 (4.2) | 28/256 (10.9) | -6.7 (-11.2 to -2.1) | 0.004** | -7.1 (-11.9 to -2.2) | 0.004** |  |
|  | Per-protocol analysis set | SBP <140 mmHg | 6/96 (6.3) | 4/78 (5.1) | 1.1 (-5.8 to 8.0) | 0.75 | 2.1 (-7.7 to 11.9) | 0.67 | 0.04* |
|  |  | SBP ≥140 mmHg | 8/187 (4.3) | 25/213 (11.7) | -7.5 (-12.7 to -2.3) | 0.005** | -7.7 (-13.1 to -2.3) | 0.005** |  |

Abbreviation: CI, confidence interval; DAPT, dual antiplatelet therapy; END, early neurological deterioration; mRS, modified Rankin Scale; N/A, not applicable. NIHSS, National Institute of Health Stroke Scale; SBP, systolic blood pressure;

^a^ In the full analysis set population, there were 204 patients in the SBP <140 mmHg subgroup and 515 patients in the SBP ≥140 mmHg subgroup. In the per-protocol analysis set population, there were 174 patients in the SBP <140 mmHg subgroup and 400 patients in the SBP ≥140 mmHg subgroup.

^b^ Adjusted for covariates compared between DAPT and alteplase treatment groups with *P* value < 0.1 in each subgroup.

^c^ END was defined as an increase between baseline and 24 hours of 2 on the NIHSS score, but not as a result of cerebral hemorrhage.

* *P* value <0.05 and ** *P* value <0.01; *P* _int_ value means the *P* value for interaction.

# **Figure S1 Flow Chart.**


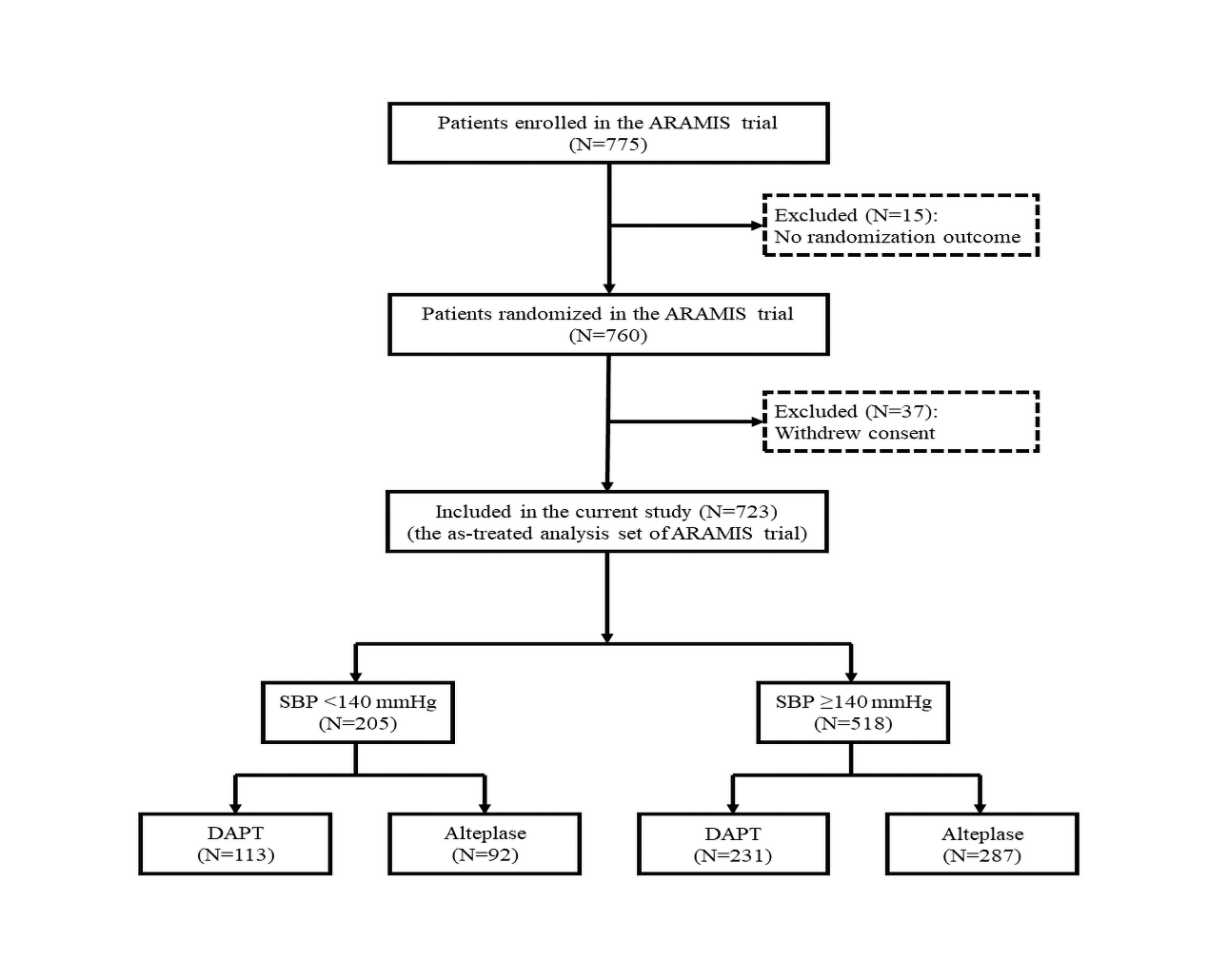


ARAMIS: Antiplatelet vs. R-tPA for Acute Mild Ischemic Stroke; DAPT: dual antiplatelet therapy; SBP: systolic blood pressure.

# **Figure S2 Subgroup Analysis.**


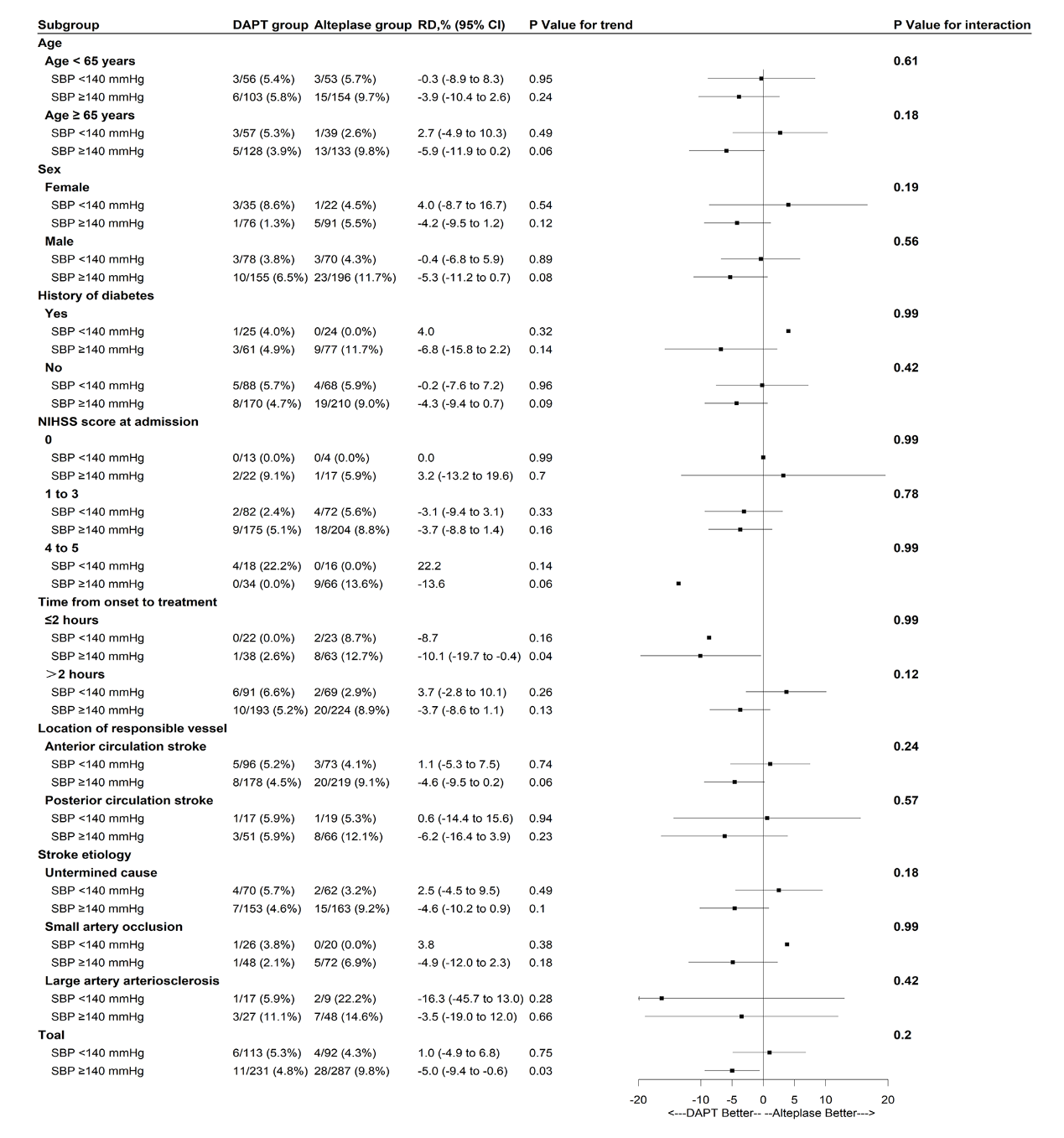


DAPT: dual antiplatelet therapy; NIHSS: National Institute Health of Stroke Scale; SBP: systolic blood pressure.
